# Supplementary material for: Temporal phase unwrapping using deep learning
Source: Sci Rep. 2019 Dec 27;9:20175. doi: 10.1038/s41598-019-56222-3 (PMC6934795; doi:10.1038/s41598-019-56222-3)
Supplement: Supplementary file 1 — Temporal phase unwrapping using deep learning: supplementary information [file 41598_2019_56222_MOESM1_ESM.pdf]

Temporal phase unwrapping using deep learning: supplementary information

Wei Yin[1,2,3], Qian Chen[1,2,7], Shijie Feng[1,2,3], Tianyang Tao[1,2,3], Lei Huang[4], Maciej Trusiak[5], Anand Asundi[6], and Chao Zuo[1,2,3,*]

***Abstract*— This document provides supplementary information for "Temporal phase unwrapping using deep learning".**

I. ARCHITECTURE OF THE DEEP NEURAL NETWORK

The whole framework of our proposed method is composed of three key steps: data process (wrapped phase recovery), phase unwrapping based on deep neural network, and phase-to-height mapping, as shown in Fig. 1 of the manuscript. The deep neural network, consisting of convolutional layers, pooling layers, residual blocks, upsampling blocks, and concatenate layer, is used to predict the fringe order map $k_h(x, y)$ from input data ($\Phi_l(x, y)$ and $\phi_h(x, y)$). The architecture of the deep neural network for training temporal phase unwrapping is depicted in Fig. 1. Among these layers and blocks, the convolutional layer and the pooling layer are common in the convolutional neural network. The size of all the kernels (or filters) used throughout the networks convolutional layers is 3×3. The residual block, making up of two convolutional layers and ReLU as shown in Fig. 1 by proposed He et al. [1], is the basic block of the residual network and perform a shortcut operation between the input tensor and two convolutional layers, which can speed up the convergence of deep networks and improve the network capability by adding layers with considerable depth. The ReLU is an activation function as follows:

$$ReLU(x) = max(0, x). \tag{1}$$

And then we introduce the multi-scale pooling layer to downsample the input tensors, which can compress and extract the main features of the tensors for the reduction of computation complexity and the prevention of overfitting. In the first path of the deep neural network, since the phase images are successively processed by one convolutional layer, a group of residual blocks, and another convolutional layer without any pooling layer and upsampling block, it keeps the tensor data in the original size of input data. On the contrary, the remaining three paths provide sparse solutions in the image plane due to the pooling operation with different scales. Therefore, in order to against the effect of pooling layers with different scales, different numbers of upsampling blocks will be required to make the sizes of the tensors in the paths uniform. The upsampling block, including a convolutional layer, a ReLU, and a sub-pixel layer as shown in Fig. 2 by proposed Shi et al. [2], is applied for the image and video super-resolution and can learn an array of upscaling filters to upscale the final low-resolution feature maps of each path into the high-resolution output instead of using bicubic interpolation. After the feature tensors of the four paths are gathered, as an important operation without learnable weights, the concatenate layer is applied for the feature combination on channel axis. And then one convolutional layer with 200 kernels makes the final prediction of the network based on the output of the concatenate layer. Due to the whole optimized design of the network, the proposed network has a total of approximately 1.4 million learnable parameters, which make high-performance TPU possible.

II. EXPERIMENTIAL SETUP AND DATA PROCESS

To prepare datasets for the deep neural network, a common FPP system is set up including a monochrome camera (Basler acA640-750um with the resolution of 640×480) and a DLP projector (LightCrafter 4500Pro with the resolution of 912×1140) in Fig. 3. In our experiments, the three-step phase-shifting fringe patterns

[1]School of Electronic and Optical Engineering, Nanjing University of Science and Technology, No. 200 Xiaolingwei Street, Nanjing, Jiangsu Province 210094, China

[2]Jiangsu Key Laboratory of Spectral Imaging & Intelligent Sense, Nanjing University of Science and Technology, Nanjing, Jiangsu Province 210094, China

[3]Smart Computational Imaging (SCI) Laboratory, Nanjing University of Science and Technology, Nanjing, Jiangsu Province 210094, China

[4]Brookhaven National Laboratory, NSLS II 50 Rutherford Drive, Upton, New York 11973-5000, United States

[5]Institute of Micromechanics and Photonics, Warsaw University of Technology, 8 Sw. A. Boboli Street, Warsaw 02-525, Poland

[6]Centre for Optical and Laser Engineering (COLE), School of Mechanical and Aerospace Engineering, Nanyang Technological University, Singapore 639798, Singapore

[7]e-mail: chenqian@njust.edu.cn

*Corresponding author: zuochao@njust.edu.cn and surpasszuo@163.com

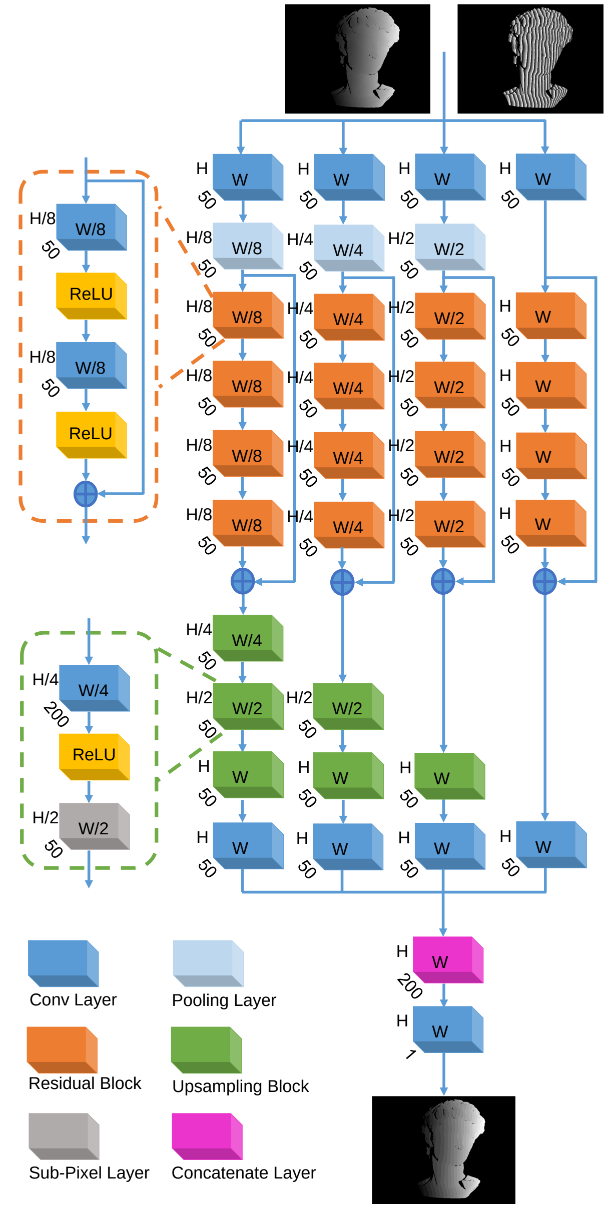

Fig. 1: Detailed architectures of the deep neural network for training temporal phase unwrapping.

with different frequencies (including 1, 8, 16, 32, and 64) are sequentially projected on the surfaces of multiple samples and synchronously captured by the camera. According to Eqs. (2) - (7) of the manuscript, due to the multiple use of MF-TPU, the wrapped phases and the corresponding $k_h(x, y)$ with different frequencies can be correctly acquired to create the training data, the verification data, and the test data. Aiming at phase unwrapping for different $\phi_h(x, y)$, the proposed network will be trained using the different dataset (including the single-period phases $\Phi_l(x, y)$, $\phi_h(x, y)$ and $k_h(x, y)$ with the corresponding high frequency), which are di-

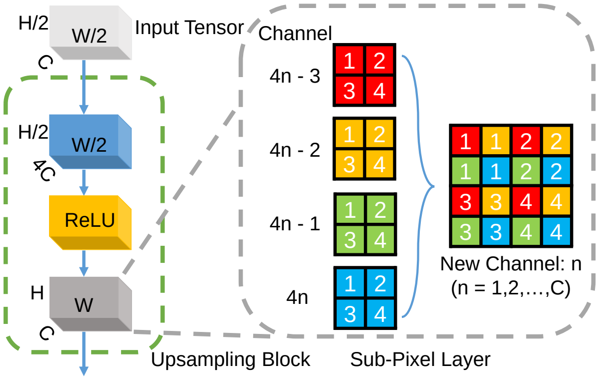

Fig. 2: Architectures of the upsampling block and the sub-pixel layer.

vided into 800 image pairs for training, 200 image pairs for validation, and 200 image pairs for testing. These data need to be preprocessed before training the deep neural network. Since the images captured by the camera contains the background and the measured object, the background can be removed by the following formula:

$$\begin{aligned} B(x,y) &= \frac{1}{2}\sqrt{[B_1(x,y)]^2 + [B_2(x,y)]^2}, \\ Mask_v(x,y) &= B(x,y) > Thr1, \\ B_1(x,y) &= \sum_{n=0}^{2} I_n^c(x,y)\sin\frac{n\pi}{2}, \\ B_2(x,y) &= \sum_{n=0}^{2} I_n^c(x,y)\cos\frac{n\pi}{2}, \end{aligned} \tag{2}$$

where $B(x, y)$ is the intensity modulation of $I_n^c(x, y)$, $Thr1$ is the preset threshold to distinguish the object from the low-modulation background. After thresholding, the valid measurement points labelled by $Mask_v(x, y)$ are further used for network training and 3D reconstruction, It should be noted that the threshold $Thr1$ should be adjusted for object surfaces with different reflectivities. In most cases, $Thr1$ is set as 0.01 for various objects in our experiments. The proposed network is implemented using TensorFlow framework (Google) and is computed on a GTX Titan graphics card (NVIDIA). In the network configuration, the loss function is set as mean square error (MSE), the optimizer is $Adam$, the size of mini-batch is 2, and the training epoch is set as 300. To avoid over-fitting as the common problem of the deep neural network, $L2$ regularization is adopted in each convolution layer of residual blocks and upsampling blocks instead of all convolution layers of the proposed network, which can enhance the generalization ability of the network.

After training at 300 epoch took about 10 hours, the losses of the training and validation dataset are shown

as in Fig. 4. It can be drew a conclusion from Fig. 4 that the train loss and validation loss of models for TPU with different high frequency are both reduced significantly due to the optimized design of the network and data process which contains choosing $k_h(x, y)$ as the network's label and the background removal operation. Besides, the overfitting problem has been slightly mitigated by comparing Figs. 4 (a) and 4 (b). This result indirectly reveals that our method can provide better phase unwrapping results and even directly and reliably recover the absolute phase with 64 periods from one unit-frequency phase.

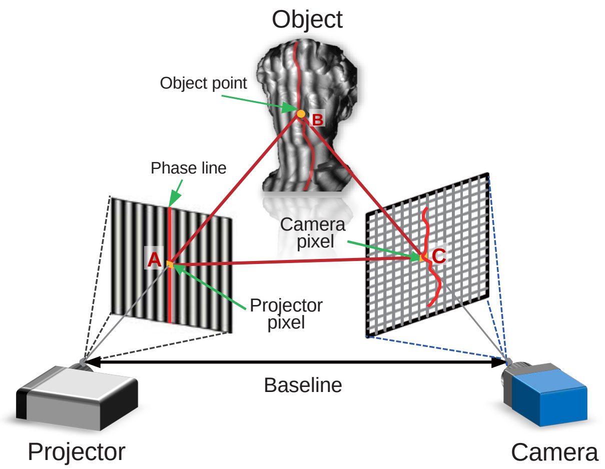

Fig. 3: Schematic of the FPP system for 3D measurements.

III. SPECIFIC OPERATION OF THE DEEP NEURAL NETWORK

It is often acknowledged that deep learning models are like "black boxes". It is difficult for the public to understand how deep learning works and why their performance is so good. Though this view may be partially correct for some types of deep learning models, the truth is quite different for convolutional neural networks. The representations of data learned by convolutional layers are highly amenable to visualization, which is largely because they are representations of visual concepts. With the rapid development of convolutional neural networks, various techniques have been developed to visualize and interpret these representations [3]. For the purposes of this supplementary information, we will not investigate all of them, but we will introduce some of the most accessible and useful visualization tools to reveal the specific operational behavior of our trained network.

In the first two subsections, we have introduced in detail that how to design a deep neural network for phase unwrapping and how to prepare the dataset for training

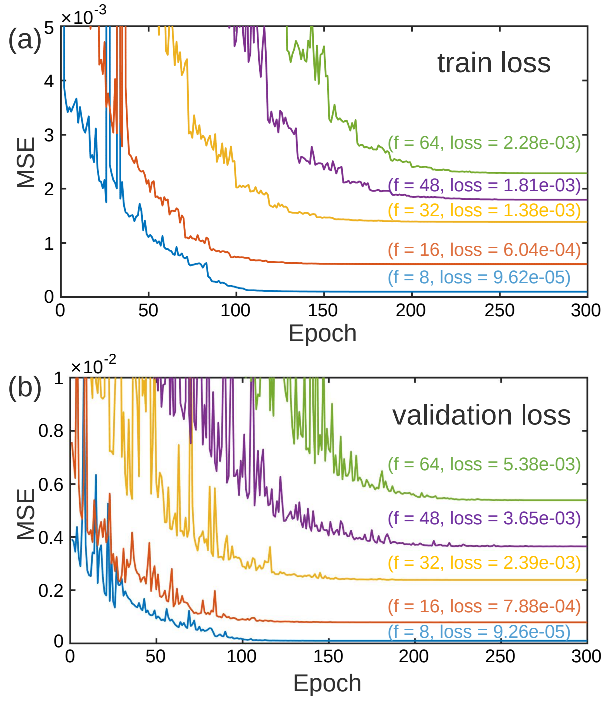

Fig. 4: Loss curves of the training and validation dataset for the proposed neural networks. (a) Loss curves of the training dataset. (b) Loss curves of the validation dataset.

the proposed network. So we already know the input, output, and the entire operating flow of the network. As a practical case, the phase unwrapping result for a sample of the testing dataset is shown in Fig. 5. It can help to discover the specific operation of every layer in a bottom-up manner by checking the operation of the last convolution layer. 200 feature maps of the four paths collected by the concatenate layer will directly serve as the input of the last convolution layer as shown in Fig. 6. As described in Supplementary Section 1, without using any pooling layer and upsampling block, these layers from the first path of the deep neural network output 50 feature maps, some of which are similar in structure and content to the final predictions of the network. In contrast, 150 feature maps from the other three paths will be treated as sparse solutions in the image plane due to the pooling operation with different scales. The operation of the last convolution layer can be formulated as:

$$p = \sum_{i=0}^{199} f_i * w_i + b, \tag{3}$$

where f_i is the i-th input feature map, w_i is a learnable 2D filter kernel with a size of 3×3, $*$ refers to the convolution operation, b is a bias, and p is the output. Due to a large number of convolution operations involved, it is difficult to visually observe the specific operation

of the last convolution layer on the input feature map from the weight matrix. Therefore, we try to find the calculation relationship between the input feature map and the weight in the frequency domain which can be expressed as

$$G_i = F_i \cdot W_i, \tag{4}$$

where F_i and G_i are the Fourier transform of the i-th input feature map and $f_i * w_i$, W_i is the Fourier transform of a filter kernel (i.e., the transfer function). The first 50 weight matrices from the last convolutional layer of the network are extracted and transformed into transfer functions by the zero-padding operation on the spatial domain as shown in Fig. 7. It can be deduced from Figs. 6 and 7 that the final result predicted by the network is mainly composed of some feature maps similar in structure and content. So the next step is to analyze the relationship between these feature maps and the final prediction result, such as the 26th input feature map f_{26} and the result $Prediction$ as shown in Figs. 8 (a) and (c). We take one cross-section on f_{26} and $Prediction$ to present the comparison results in Fig. 8 (d). Obviously, the change regulation of f_{26} is close with $Prediction$ but the value is almost half of $Prediction$. Then f_{26} is implemented with some simple transformation operations according to the following formula:

$$g_{26} = ceil(2 \times (f_{26} * w_{26})), \tag{5}$$

where $ceil()$ is an upward rounding function, g_{26} and the corresponding comparison are both shown in Figs. 8 (b) and (e). Although Eq. (5) is an empirically driven formula, it can be strongly proved from this comparison result that the 26th feature map has a high correlation with the prediction results. Undoubtedly, the feature maps from the first path mainly constitute the low-frequency component of the prediction result. Since the pooling operation extends the receptive domain of convolution layers, 150 feature maps from the other three paths represent the high-frequency components of the prediction result as auxiliary information and make results of the first path high-quality. Different from previous works, the results present in this supplementary information that we reveal for the first time the specific operation of the network for phase unwrapping.

IV. THE COMPENSATION OPERATION FOR FRINGE ORDER ERRORS

In the first experiment of the manuscript, it can be find that the fringe order errors are mostly concentrated on the dark regions and object edges where the fringe quality is low. Different from MF-TPU, phase unwrapping errors caused by the low signal-to-noise ratio (SNR) region of phases is significantly reduced by

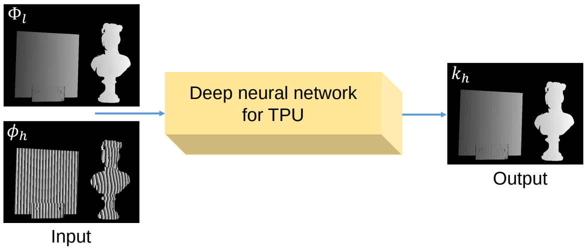

Fig. 5: The phase unwrapping result for a sample of the testing dataset.

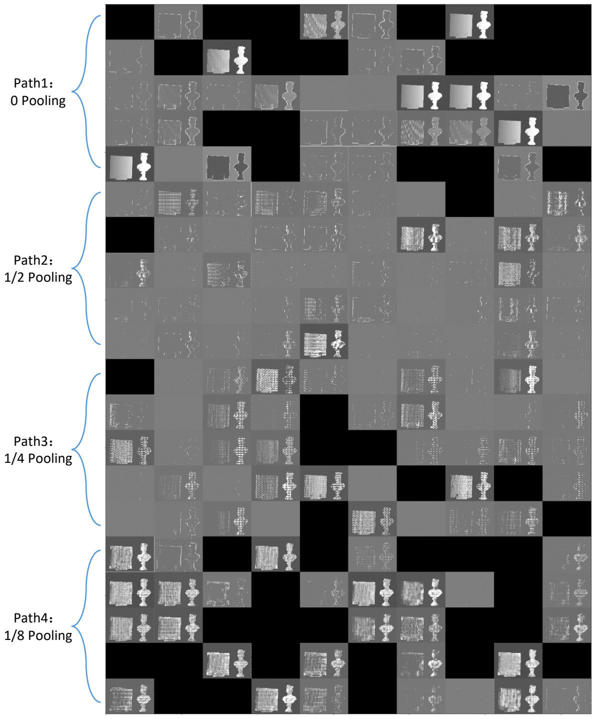

Fig. 6: The input of the last convolution layer for a sample of the testing dataset.

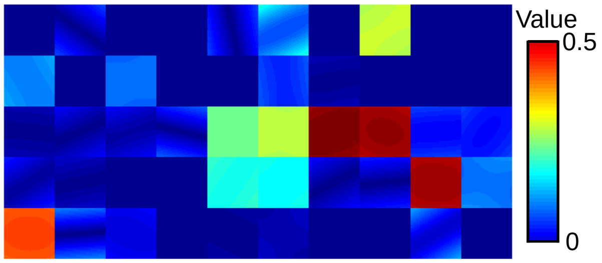

Fig. 7: The transfer function maps for first 50 filter kernels of the last convolution layer in the deep neural network.

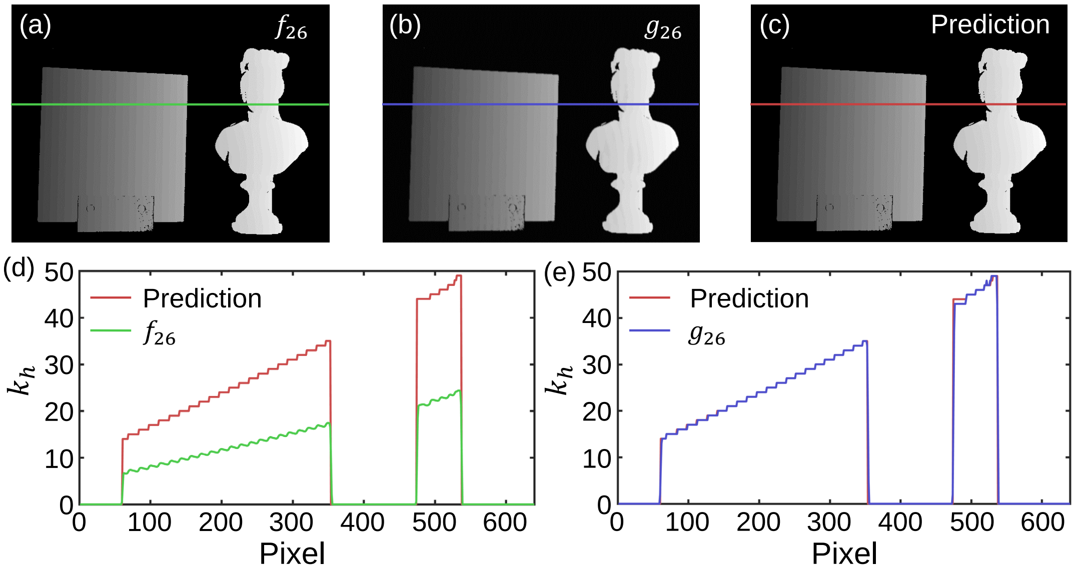

Fig. 8: Comparison results between f_{26}, g_{26}, and $Prediction$. (a) The 26-th input feature map f_{26}; (b) g_{26} obtained according Eq. (5); (c) The prediction result $Prediction$; (d) The comparison results between f_{26} and $Prediction$; (e) The comparison results between g_{26} and $Prediction$;

using DL-TPU. For these low SNR region, the remaining phase errors have the characteristics of accumulation and can be easily further corrected by some compensation algorithm for fringe order errors [4]–[6]. For the simplest case, it is common that the median filter can be applied to effectively reduce phase unwrapping errors of MF-TPU. But it still cannot remove and correct error points completely using median filters of different sizes (including 3×3, 5×5, and 7×7) as shown in Figs. $9(a) - (d)$. Although the neural network also involves of several convolution kernels (the size is 3×3 in DL-TPU) essentially, it can achieve much better phase unwrapping performance due to a large number of convolution operation on the multi-scale path in Fig. $9(e)$. Consequently, the trained models can substantially decrease error points to provide better phase unwrapping results (even $f_h = 64$) and lower error rates, which demonstrates the capability and reliability of DL-TPU for phase unwrapping.

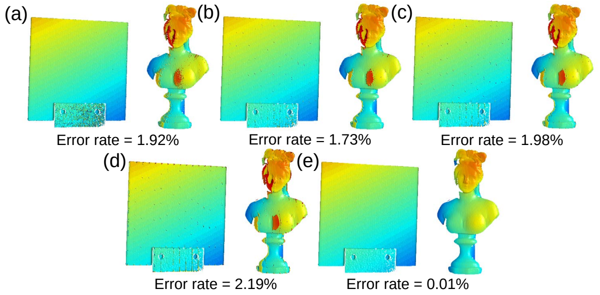

Fig. 9: Comparison of the 3D reconstruction results after phase unwrapping for a sample on the testing dataset using MF-TPU, MF-TPU with median filters, and DL-TPU. (a) The 3D results of MF-TPU. (b)-(d) The 3D results of MF-TPU with median filters of different sizes including 3×3, 5×5, and 7×7. (e) The 3D results of DL-TPU.

REFERENCES

[1] K. He, X. Zhang, S. Ren, and J. Sun, "Deep residual learning for image recognition," in *Proceedings of the IEEE Conference on Computer Vision and Pattern Recognition*, 2016, pp. 770–778.

[2] W. Shi, J. Caballero, F. Huszár, J. Totz, A. P. Aitken, R. Bishop, D. Rueckert, and Z. Wang, "Real-time single image and video super-resolution using an efficient sub-pixel convolutional neural network," in *Proceedings of the IEEE Conference on Computer Vision and Pattern Recognition*, 2016, pp. 1874–1883.

[3] F. Chollet, *Deep learning with python*. Manning Publications Co., 2017.

[4] D. Zheng, F. Da, Q. Kemao, and H. S. Seah, "Phase-shifting profilometry combined with gray-code patterns projection: unwrapping error removal by an adaptive median filter," *Opt. Express*, vol. 25, no. 5, pp. 4700–4713, 2017.

[5] C. Zuo, T. Tao, S. Feng, L. Huang, A. Asundi, and Q. Chen, "Micro fourier transform profilometry (μftp): 3d shape measurement at 10,000 frames per second," *Opt. Lasers Eng.*, vol. 102, pp. 70–91, 2018.

[6] W. Yin, S. Feng, T. Tao, L. Huang, M. Trusiak, Q. Chen, and C. Zuo, "High-speed 3d shape measurement using the optimized composite fringe patterns and stereo-assisted structured light system," *Opt. Express*, vol. 27, no. 3, pp. 2411–2431, 2019.
